# MOTIVATIONS TO VOLUNTEER: SURVEY OF A SERVICE AGENCY IN RURAL MICHIGAN

**DOI:** 10.1093/geroni/igac059.1984

**Published:** 2022-12-20

**Authors:** Rita Hu, Ruth Dunkle, Julie Tarr

**Affiliations:** University of Michigan, Ann Arbor, Michigan, United States; University of Michigan, Ann Arbor, Michigan, United States; ShareCare of Leelanau, Leland, Michigan, United States

## Abstract

While volunteers are the foundation of service agencies in rural communities, they are difficult to recruit. This study aims to understand the characteristics of volunteers in a rural community that could aid in recruitment. All volunteers (N=127) for an organization providing services to keep older adults in their homes in rural Michigan were approached to participate in the study. Data were gathered via telephone-interview survey (N=76) and in-person focus groups (N=14) in the summer of 2021 to understand demographic factors as well as benefits and community factors contributing to volunteer participation. Most volunteers were female (61%), married (71%), over age 70 (M=71), and lived with others (72%). In the past year, 49% volunteered 1-5 hours per week and with multiple organizations (70%). They identified the primary benefits from volunteering as getting to know new people (66%). Volunteers agreed that they feel they contribute to the community (97%), have a strong attachment to the community (91%), and have the ability to make a difference in the community (95%). Volunteers were less aware of what could be done to meet the needs in the community (54%) and understand the needs and problems facing the community (59%). Focus group data suggest that volunteers desire information about community needs and aging in place. Due to the lack of centralized locations, social and educational hot spots such as coffee shops could attract new volunteers. Having a sense of community is an important component of volunteering and should be fostered when recruiting new volunteers.

